# Hand Passive Mobilization Performed with Robotic Assistance: Acute Effects on Upper Limb Perfusion and Spasticity in Stroke Survivors

**DOI:** 10.1155/2017/2796815

**Published:** 2017-09-28

**Authors:** Massimiliano Gobbo, Paolo Gaffurini, Laura Vacchi, Sara Lazzarini, Jorge Villafane, Claudio Orizio, Stefano Negrini, Luciano Bissolotti

**Affiliations:** ^1^Department of Clinical and Experimental Sciences, University of Brescia, Brescia, Italy; ^2^Laboratory of Clinical Integrative Physiology, University of Brescia, Brescia, Italy; ^3^Laboratory of Neuromuscular Rehabilitation, Teresa Camplani Foundation, Brescia, Italy; ^4^IRCCS Don Gnocchi Foundation, Milan, Italy; ^5^Functional Rehabilitation Service, Teresa Camplani Foundation, Brescia, Italy

## Abstract

This single arm pre-post study aimed at evaluating the acute effects induced by a single session of robot-assisted passive hand mobilization on local perfusion and upper limb (UL) function in poststroke hemiparetic participants. Twenty-three patients with subacute or chronic stroke received 20 min passive mobilization of the paretic hand with robotic assistance. Near-infrared spectroscopy (NIRS) was used to detect changes in forearm tissue perfusion. Muscle tone of the paretic UL was assessed by the Modified Ashworth Scale (MAS). Symptoms concerning UL heaviness, joint stiffness, and pain were evaluated as secondary outcomes by self-reporting. Significant (*p* = 0.014) improvements were found in forearm perfusion when all fingers were mobilized simultaneously. After the intervention, MAS scores decreased globally, being the changes statistically significant for the wrist (from 1.6 ± 1.0 to 1.1 ± 1.0; *p* = 0.001) and fingers (from 1.2 ± 1.1 to 0.7 ± 0.9; *p* = 0.004). Subjects reported decreased UL heaviness and stiffness after treatment, especially for the hand, as well as diminished pain when present. This study supports novel evidence that hand robotic assistance promotes local UL circulation changes, may help in the management of spasticity, and acutely alleviates reported symptoms of heaviness, stiffness, and pain in subjects with poststroke hemiparesis. This opens new scenarios for the implications in everyday clinical practice. Clinical Trial Registration Number is NCT03243123.

## 1. Introduction

Stroke represents the most common cause for adult upper limb (UL) motor impairments [1], leading for almost the 80% of hand motor function disorders as a consequence of hemiplegia [2–4]. Motor recovery is frequently poor or insufficient, with only one-third of stroke patients regaining dexterity within the first six months [5]. Less than 45% of stroke patients are likely to achieve complete functional recovery, while the majority of this population will reveal a variable degree of residual impairment and inability to accomplish daily life activities [6–9]. Beside motor control and sensory deficits, stroke survivors present common complications such as pain, spasticity, joint constraint, and skin or vascular damage, which represent paramount challenges in stroke management [10, 11].

To counteract these problems and to help in restoring/improving upper and lower limb function, a wide range of technically advanced devices designed to assist physical rehabilitation are increasingly at disposal for therapists. The robot-assisted therapy is one of the most innovative and promising approaches intended to recover function after stroke [12–15]. Robotic devices assist patients in performing repetitive tasks (active or passive exercises), addressing several poststroke rehabilitation purposes (e.g., functional training; joint flexibility maintenance and joint stiffness reduction; prevention of muscle/tendon shortening with related deformities, pain, and alterations in muscle-tendon unit mechanics; enhancement of somatosensory and proprioceptive input; reduction of edema, deep venous thrombosis, decubitus ulcers, and pain by promoting circulation) [11, 16–19].

Against this background, the assessment of the specific biological effects and mechanisms of currently available robot-assisted interventions is paramount to achieve the best clinical outcomes and to achieve proper clinical decision making, by helping clinicians in choosing the most appropriate modality of intervention for each patient and condition.

The primary purpose of the study was to evaluate the immediate effects of repetitive, robot-assisted hand passive motion on forearm local perfusion, as well as the acute effects on UL spasticity in subjects with poststroke hemiparesis. For a more comprehensive view, we aimed at assessing also subjects' perception of UL heaviness, stiffness, and pain, since these symptoms can be a further barrier to active movement and may therefore affect substantially the effectiveness of rehabilitation protocols [20]. By obtaining more insight into the acute effects of a single-session intervention, second aim of the study was to depict some possible implications of robotic rehabilitation in terms of protocol management within everyday clinical practice.

## 2. Methods

### 2.1. Overview

A single arm, pre-post study was conducted with a pragmatic approach (i.e., in real-life routine practice conditions [21]). The institutional Ethical Committee approved the experimental protocol. Informed consent was obtained from all participants according to the Declaration of Helsinki.

### 2.2. Participants

Patients enrolment was conducted within a Physical Therapy Department during a period of 4 months. Inclusion criteria were first event of cerebrovascular stroke; unilateral paresis; ability to remain in a sitting posture. Exclusion criteria were bilateral impairment; cognitive or behavioural dysfunction that would compromise the experiment execution; finger flexion contracture; DeQuervain's tenosynovitis; degenerative or nondegenerative neurological conditions in which pain perception could be altered; refusal or inability to provide informed consent. The diagnosis of stroke was performed by computed tomography or magnetic resonance imaging scan.

### 2.3. Intervention

**Figure 1:**
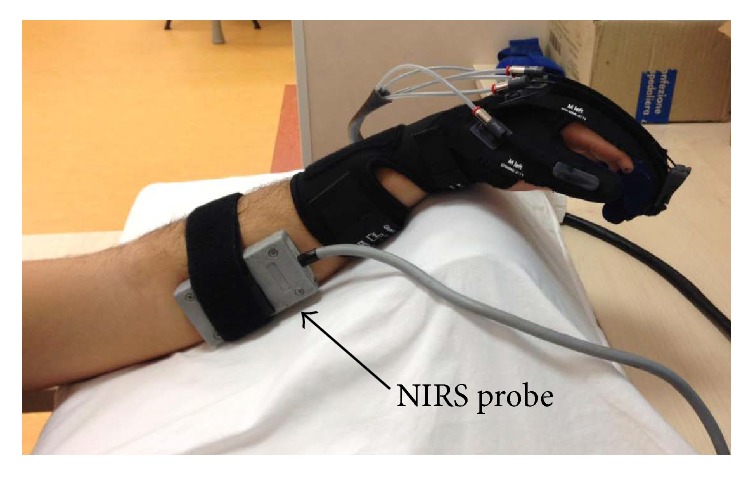
Experimental setup. The Gloreha glove was applied on the paretic hand. The NIRS probe (grey box) was applied over the forearm ventrolateral surface.

The intervention was performed using the Gloreha (Idrogenet, Italy) robotic system. Among the robotic devices providing physical therapy, Gloreha is an active device designed for motion assistance, with a double version for both hospitals/rehabilitation centres (professional version) and patients home-based use (low-cost version) [1]. It consists of a soft exoskeleton similar to a glove that envelops wrist and fingers of the paretic hand with Velcro® attachments and straps (see Figure 1). The setup takes less than 5 minutes, in accordance with the recommendations by Dijkers et al. (1991) [22]. Passive joint mobilization is provided by a hydraulic system that generates forces transmitted to the fingers through semi-rigid cables (Serpelloni et al., 2016 [23], for technical details). The software allows selecting different passive exercises and ranges of motion. Its innovative system enables calibrated sequential movement of each individual finger or combined finger motion during the simultaneous observation of a 3D model on a screen that reproduces the movement generated by the glove in real time.

In this study, each patient underwent a single session of robot-assisted passive mobilization of the paretic hand. The treatment session lasted 20 minutes and consisted of repetitive passive exercises provided in different modalities:* isolated* (sequential flexion and extension of each finger individually; duration = 6.5 min),* pinch* (I and II fingers mobilized to produce thumb opposition and pinching; duration = 3.5 min), and* synchronous* (II-III-IV-V fingers flexed and extended simultaneously, the thumb individually, in order to perform grasping and other movements involving all fingers; duration = 10 min).

### 2.4. Outcome Measures

We used the near-infrared spectroscopy (NIRS) to evaluate the dynamics of forearm perfusion during the aforementioned different modalities of passive mobilization with the Gloreha glove. NIRS is a noninvasive technique that provides information on the changes of oxygenated haemoglobin (O_2_Hb) and deoxygenated haemoglobin (HHb) in the tissue beneath the probe (optode assembly). The total haemoglobin amount (O_2_Hb + HHb; THb) is related to regional blood flow [24, 25].

**Figure 2:**
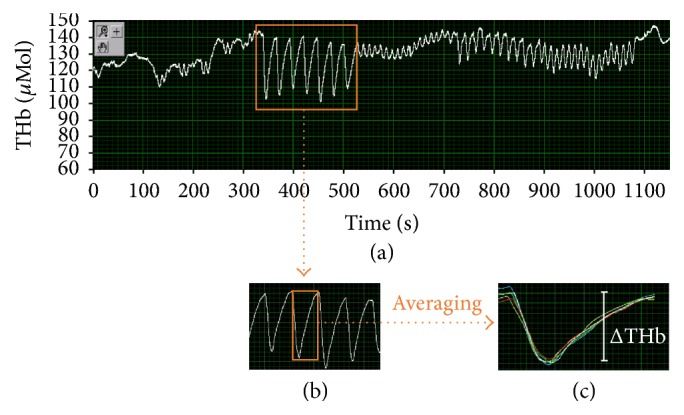
Signal analysis of the total haemoglobin (THb) profile. Panel (a) shows the THb profile acquired during a whole treatment session from a representative subject. Time windows corresponding to the duration of each passive movement modality were selected (panel (b)). For each time window, the values corresponding to the largest variation of THb dynamics were averaged (panel (c)) in order to obtain the delta value (ΔTHb) for each of the administered treatment modalities.

We employed the NIMO system (Nirox Optoelectronics, Italy) with the probe secured to the skin surface over the flexor muscles of the forearm (see Figure 1) and covered with an optically dense sheet to minimize intrusion of ambient light. Measurement depth was 2.5 cm. NIRS parameters were acquired during the whole 20 min treatment session with a sampling frequency of 40 Hz. Values corresponding to the largest variation of THb dynamics during robot-assisted mobilization were quantified and averaged; the THb baseline data (i.e., average value of a 30-second rest just before the Gloreha session) was then subtracted to the averaged largest variation in order to obtain the delta value (ΔTHb) for each of the administered passive movement modalities (see Figure 2).

### 2.5. Functional Assessment

In order to evaluate patient's acute response to treatment, clinical outcomes were measured before (pre) and during the 5–15 minutes following (post) the intervention by trained assessors.

The Modified Ashworth Scale (MAS) was employed as primary outcome to measure UL spasticity [26]. MAS tests joint resistance to passive movement with varying degrees of velocity within a resulting range from 0 to 4, with 6 choices (a score of 0 indicates “no increase in muscle tone” while 4 refers to “joint rigidity”) [27]. Assessment of spasticity included shoulder abduction, elbow extension, supination, wrist extension, and fingers extension movements with the patient in resting position. Previous studies on poststroke suggest a MAS score ≥ 1 for any of the performed passive movements as an indicator of the presence of spasticity [28].

Self-report measures of perceived heaviness, joint stiffness, and pain for shoulder, elbow, and hand (wrist and fingers) regions were used as secondary outcomes; data were collected and quantified by using a numeric rate scale with values ranging from 0 (absence of the investigated symptoms) to 100 (worst possible sensation).

### 2.6. Statistical Analysis

Data were analysed using SigmaPlot 12 (Systat Software Inc., USA). Effects were expressed as mean differences ± standard deviations (SD). For THb changes, a one-way analysis of variance (ANOVA) with repeated measures was used to evaluate significant differences between baseline and the three modalities of intervention* (isolated; pinch; synchronous)*. For MAS and self-report measures, a paired *t*-test was performed to test significant differences between pre- and posttreatment conditions. When the distribution of values did not pass the normality test, the Wilcoxon Signed Ranks test was used. Significance was set at *p* < 0.05.

## 3. Results

The inclusion criteria were met by 23 outpatients (13 males; 10 females), aged 60.4 ± 13.2 years (range: 40–84 years). Twelve participants had chronic stroke (>6 months; range: 9 to 508 months), while 11 patients were in subacute poststroke phase (≤6 months; range: 3 to 6 months); 15 subjects had ischemic stroke, while the remaining 8 had a haemorrhagic stroke. Mini-Mental State Examination score [29] was ≥24 for all participants. The arm score of the Motricity Index [30] was 37.7 ± 22.9 (subset scores: pinch grip = 10.4 ± 9.8; elbow flexion = 14.0 ± 8.8; shoulder abduction = 13.3 ± 7.9).

Forearm perfusion changed during hand passive motion. In particular, ΔTHb was 2.53 ± 1.80 *μ*Mol during the* isolated* modality, 2.03 ± 1.19 *μ*Mol during the* pinch* modality, and 5.42 ± 1.78 *μ*Mol during the* synchronous* modality. Furthermore, with respect to baseline, ΔTHb significantly improved (*p* = 0.014) during the* synchronous* modality.

MAS scores and self-report measures for pre- and postconditions are reported in Table 1. MAS scores decreased globally, with statistically significant changes for the wrist (*p* = 0.001) and fingers (*p* = 0.004). Subjects reported decreased symptoms of UL heaviness and stiffness after treatment, especially for the hand area. Among the 23 participants, only 6 patients reported UL pain; 4 of them reported decreased pain after the intervention, while the other 2 reported no changes in the perceived pain level.

## 4. Discussion

Our study supports novel evidence that hand robotic assistance promotes local UL circulation, acutely alleviates reported symptoms of heaviness, stiffness, and pain in subjects with poststroke hemiparesis, and may contribute in the management of spasticity after stroke.

### 4.1. Circulatory Adaptations

Tissue perfusion of the forearm, assessed through quantification of the changes in THb within the flexor muscles, changed significantly during the* synchronous* modality of the intervention. This phenomenon suggests a beneficial effect of robot-assisted therapy in terms of improved regional blood flow changes during passive movements involving all fingers simultaneously which may, in turn, facilitate the washout of catabolites and pain-related molecules accumulating in the tissues of poorly active upper limbs and thus possibly alleviate regional pain [31, 32].

A further consideration pertains to angiogenesis induced by passive movement. Previous studies using the “passive movement model” have clearly demonstrated that passive motion, although less potent than muscle contraction, may induce an increase in blood flow and muscle stretch which, in turn, elicit mechanical signals that initiate angiogenesis in skeletal muscles [33–36]. It has to be underlined that NIRS detects variations in haemoglobin amounts specifically at the microcirculation level and it is therefore able to monitor muscle capillary supply [24, 25]. On these bases, since repetitive improved capillary supply represents an important mechanical factor for angiogenesis due to the shear stress phenomenon following increased flow, our data suggest that the* synchronous* modality of finger passive motion with robotic assistance may have important clinical implications for muscle tissue function and circulatory homeostasis, in light of the fact that low levels of mechanical impact, as in the case of hemiparesis, may promote endothelial cell apoptosis and capillary regression [36, 37].

### 4.2. Functional Effects

The study found that robot-assisted continuous passive motion of the paretic hand acutely alleviated UL spasticity, with more beneficial effects at the wrist and fingers levels. In addition, self-reported measures indicated a temporary improvement of the UL condition likely due to repetitive movements and circulation changes, as described in the previous paragraph.

The combination of abnormal muscle tone, joint stiffness, and regional pain is known to interfere with the execution of functionally useful movements and to lead to a reduction in motor activity and to loss of dexterity. On the contrary, when muscle tone is becoming more normal and pain is reduced, active motion will facilitate functional retraining [31, 38]. The alleviation of the aforementioned problems not only is therefore beneficial for the patient himself, but may bring benefits also for the therapist who, in turn, may be assisted in providing more effectively the recommended supervised exercises and manual therapy techniques. In other words, the temporarily enhanced functional status of the patient can be considered as a therapeutic “window of opportunity” for the therapist, during which she/he may be able to more optimally exploit the residual functions of the patient and attain better motor outcomes. In this line, robotic passive hand mobilization should be administered before active engagement, in order to improve temporarily the UL functional status and facilitate the subsequent active training with the therapist. These considerations could be taken into account when managing the sequence of therapeutic interventions provided to patients during the day, with the purpose of designing more effective rehabilitation protocols.

### 4.3. Study Limitations

Due to the chosen experimental design and the pragmatic characteristics of the study, the present work is not free of some intrinsic limitations. First, there was not a group of control to test the extent of improvement and modification given by the Gloreha glove use with respect to other modalities of passive mobilization. Second, it was not possible to treat patients each day at the same hour; this might induce a methodological limitation of the study, since vascular responses fluctuate during the day. Anyway, this confounding variable is not easy to control, as circadian patterns modulating neural and vascular functions can be profoundly altered and highly variable in stroke patients [39]. Similarly, due to the pragmatic approach of the study, previous activity during the day (e.g., manual therapy, occupational therapy, physical therapy, and recreational activity) could not be controlled and therefore patients may have undergone basal assessment, as well as treatment, under different conditions. Finally, we were not able to assess the total duration of the effects induced by the single-session intervention. For this, at the moment, only limited information on the duration of the therapeutic window can be provided.

## 5. Conclusions

Passive mobilization has several beneficial effects and represents a fundamental part of a comprehensive rehabilitation program for stroke recovery, being particularly important when the patient does not have the physical or cognitive ability to actively move the extremity. Poor activity may be due also to the presence of spasticity, joint stiffness, and pain which often interfere with the rehabilitation program and impede functional motor recovery. The present findings provide evidence that hand passive mobilization with robotic assistance has the ability to concurrently enhance local circulation and acutely alleviate UL spasticity, stiffness, and pain. The temporarily ameliorated UL condition could be exploited by therapists in everyday clinical practice to improve the effectiveness of active training when performed during the therapeutic window of opportunity induced by previous robot-assisted passive motion. The hypotheses discussed above, although based on established foundations and observations reported in other publications, need to be verified with further research and dedicated experimental designs.

## Figures and Tables

**Table 1:** Modified Ashworth Scale scores and self-report measures before (pre) and after (post) the intervention. Values are expressed in mean ± SD. Bold values of *p* indicate statistically significant differences.

	Pre	Post	*p* value
*Modified Ashworth Scale*			
Shoulder	0.4 ± 0.8	0.4 ± 0.9	1.000
Elbow	1.3 ± 0.6	1.1 ± 0.7	0.164
Supination	0.8 ± 0.8	0.7 ± 0.6	0.188
Wrist	1.6 ± 1.0	1.1 ± 1.0	**0.001**
Fingers	1.2 ± 1.1	0.7 ± 0.9	**0.004**
*Heaviness*			
Shoulder	46.6 ± 30.2	40.5 ± 27.3	**0.016**
Elbow	37.3 ± 26.7	34.5 ± 26.6	0.150
Hand	43.2 ± 34.0	34.8 ± 31.6	**0.027**
*Stiffness*			
Shoulder	41.8 ± 34.3	35.7 ± 31.6	0.031
Elbow	39.8 ± 32.9	32.3 ± 30.8	**0.016**
Hand	51.8 ± 30.5	40.9 ± 30.3	**0.020**
*Pain*			
Shoulder	35.0 ± 18.0	26.0 ± 15.2	0.500
Elbow	35.0 ± 18.0	25.0 ± 13.2	0.250
Hand	49.2 ± 16.3	36.7 ± 19.7	0.250
